# Prevalence and Correlates of Microalbuminuria in Children with Sickle Cell Anaemia: Experience in a Tertiary Health Facility in Enugu, Nigeria

**DOI:** 10.1155/2012/240173

**Published:** 2012-09-28

**Authors:** Christopher Bismarck Eke, Henrietta Uche Okafor, Bede Chidozie Ibe

**Affiliations:** Department of Paediatrics, University of Nigeria Teaching Hospital, Ituku/Ozalla, Enugu, Nigeria

## Abstract

Microalbuminuria is a pre-clinical marker of renal damage in children with sickle cell anaemia and can predict renal failure. Reported prevalence rates increased with age. In Nigeria, burden of disease and prevailing poor health facilities necessitate its screening, determination of prevalence and associated risk factors. It is a cross-sectional as well as descriptive study. Screening microalbuminuria used subjects' early morning urine. Socio-demographic as well as clinical details were ascertained using semi-structured questionnaires and case files. Associations and statistical relationship of prevalence rates and clinical/epidemiological data were ascertained using chi-squared and multivariate analysis (*P* < 0.05). Two hundred children with sickle cell anaemia (4–17 years) in steady state and 200 age/gender-matched controls were enrolled. Prevalence of microalbuminuria was ,respectively, 18.5% and 2.5% for subjects and controls (*P* = 0.001). Microalbuminuria was commoner in females (19.8%) than males (17.4%) *P* = 0.70, increased with age (*P* = 0.016), significantly associated with haemoglobin level (*P* = 0.002) and hospitalizations (0.001). Subjects had normal renal function. Hospitalizations and haemoglobin levels showed statistical significance on multivariate analysis. Prevalence of microalbuminuria is 18.5%. Age, haemoglobin concentrations, and higher hospitalizations influenced microalbuminuria among subjects. Screening for microalbuminuria should be incorporated in the case management of subjects with identified risk factors.

## 1. Introduction 

Sickle cell nephropathy is a major complication of sickle cell disease and results from recurrent renal vasoocclusion, ischaemia-reperfusion injury, and loss of renal mass [1]. It is characterized by glomerular hypertrophy and focal glomerulosclerosis [2, 3]. Proteinuria is one of the most common clinical manifestations of sickle cell nephropathy [4, 5]. 

Lowest level of albuminuria (20–200 mg/L) known as microalbuminuria [6] is a preclinical marker of glomerular damage predicting progressive renal failure in conditions like diabetes mellitus also associated with hyperfiltration, and hyperperfusion [7]. Microalbuminuria has been defined as an abnormally or supranormal urinary excretion of albumin in the absence of clinical proteinuria (i.e., proteinuria detectable by use of conventional dipstick like Albustix) [8]. Marshall et al. [9] defined microalbuminuria in terms of timed overnight urine collection as an albumin excretion rate greater than 20 *μ*g per minute.

Various proportional rates of microalbuminuria have been reported in children with sickle cell anaemia ranging from 18.4% to 46% [7, 10–13]. 

Microalbuminuria could be detected long before positive urine test for proteinuria using conventional dipstick like Albustix or Combi 10 Multistix screen which is sensitive to urinary protein excretion that is greater or equals to 300 mg/L [14].

Prolonged period of microalbuminuria precedes persistent proteinuria which is subsequently followed by chronic renal failure (CRF) [15] and occurs with variable frequency in the sickle cell population (4%–20%) [11]. 

The identification of risk factors for microalbuminuria may allow earlier intervention to prevent renal complications. [7] Considering the high burden of sickle cell anaemia in Nigeria with a prevalence rate of 2% in newborns [16] in a population of over 167 million [17], children with sickle cell anaemia are prone to developing proteinuria and chronic renal failure with advancing age. The attendant need for renal replacement therapy is either nonexistent or very expensive to sustain in our environment. Hence, the need to incorporate in the management of children with sickle cell anaemia, screening for microalbuminuria in those with identified associated risk factors as a marker of renal injury. Therapeutic interventions using antiproteinuric agents like angiotensin converting enzyme inhibitors could be applied in those with microalbuminuria to retard the progression of renal damage [18]. Further, hydroxyurea has been shown to decrease vasoocclusive events, which are thought to play a huge role in the pathophysiology of renal disease and is currently being used in some children with sickle cell disease [7]. 

This study sets out to ascertain the prevalence of microalbuminuria and its associated risk factors in children with sickle cell anaemia (HbSS) seen at University of Nigeria Teaching Hospital (UNTH), Enugu in comparison with their counter parts with normal haemoglobin (HbAA) pattern. 

## 2. Subjects and Methods 

The study was a cross-sectional descriptive study conducted at the University of Nigeria Teaching Hospital (UNTH), Enugu, South East Nigeria between August, 2008 and April, 2009. Enugu is the capital of Enugu state, Nigeria. Enugu state has an estimated population of 3,140,471 people from 2006 National Census [19]. The state is mainly populated by Nigerians of Igbo ethnic nationality. UNTH, Enugu serves the population in the state capital, Enugu, and its adjoining towns and villages. It also serves as a major referral centre to the surrounding health facilities in the South Eastern Nigeria and beyond. It is a 500-bed health facility. It runs two well-established sickle cell clinics which cater for paediatric and adult patients. The paediatric sickle cell clinic runs once weekly (every Monday). 

Study subjects consisted of 200 sickle cell anaemia children in steady state, that is apparently well (subjectively and objectively) without any evidence of recent infections, crises or other problems and had been well for at least 4 weeks after the last crisis [20, 21] who were recruited consecutively as they presented to the weekly sickle cell anaemia clinic of UNTH (convenience sampling method) while controls of comparable ages and matched for sex with normal haemoglobin pattern (HbAA) were recruited from primary and secondary schools in Enugu urban by multistage sampling. First a list of each of the registered public primary and secondary schools in Enugu urban were compiled to form the sampling frame. From these lists, the secondary schools were further stratified into coeducational, all boys' and all girls' secondary schools. Subsequently, two schools from each arm were selected by simple random sampling. Also, four primary schools were selected by simple random sampling. In all a total of 10 schools (four primary and six secondary schools) were selected. In the selected schools the pupils/students were then stratified according to their age, and sex. The selected primary schools also had nursery schools as part of the Universal Basic Education (UBE) from where controls less than six were selected.

The controls were then finally selected from their respective class registers using the systematic sampling method. Here, the first pupil/student was selected by simple random sampling, and every other 5th child was selected. A total of 200 school children who met the study inclusion criteria were selected as controls. 

 Excluded were children with sickle cell crises symptoms (fever, bone pain crisis) in the preceding 2 weeks, children with prior known overt proteinuria (detectable using Combi-10) or gross haematuria, including currently menstruating adolescent females, and children with comorbid conditions like hypertension, urinary tract infections, diabetes mellitus, retroviral infection, and children with any history of familial kidney disease.

Ethical approval was obtained from the Ethics and Research Committee of UNTH, Enugu while informed consent was obtained from the Ministry of Education, Enugu State and parents or care givers of the subjects and controls prior to the commencement of the study. 

A pretested semistructured and observer administered questionnaire was issued to the study population and their respective parents/caregivers/class teachers as the case may be. Information obtained and recorded in the questionnaire included age, gender, history of fever or bone pain crisis in the preceding 2 weeks, recent history of passage of blood in urine, history and number of hospital admissions following bone pain crisis in the past 24 months, and number of previous blood transfusions (subjects are usually transfused following severe anaemia or anaemic heart failure as chronic transfusion programme is not currently being practiced in our setting). 

 Social class of the study population was determined using the method proposed by Oyedeji [22]. Here, the social class of each child was determined based on the occupational status and educational attainment of the parents. The average of the four scores (two each for the father and mother, that is, occupational status and educational attainment) to the nearest whole number was the social class of each child selected in the study. According to the protocol, in cases where one of the parents is late, the social class of the child is assessed by that of the living parent or guardian. 

Subjects had clinical examination, and their weights, heights and body mass index determined. Also determined variables included the axillary temperature and pulse rate following standard protocols. Blood pressure was measured using mercury in glass sphygmomanometer (Accoson brand) with a cuff size that covered at least two-thirds of the child's right arm in a sitting position. Three different readings were taken and the average was taken as actual blood pressure. The systolic blood pressure was taken at the point when the Korotkoff sound became audible (phase I) and the diastolic blood pressure at the point at which the sound became muffled (phase IV). The readings were recorded to the nearest 2 mmHg. The blood pressure percentile (for systolic and diastolic blood pressure, resp.) for each selected child was read off from the fourth report on diagnosis, evaluation, and Treatment of high blood pressure in children aged 1 to 17 years using the gender and age appropriate charts. Hypertension was defined as average systolic blood pressure that was greater than or equals to the 95th percentile for sex, age and height [23]. All enrolled subjects and controls were issued with well-labeled universal urine bottles for the collection of 10 millilitre of early morning urine. Children and their care-givers were instructed on how to collect their early morning midstream urine and clean catch urine for subjects less than six years on the day of their appointment after first washing of their hands. Venous blood samples were collected from all participants to confirm their haemoglobin genotype by electrophoretic method as well as determine their serum creatinine and haemoglobin concentrations. The controls with AS genotype from the results were counseled and excluded from the study. The glomerular filtration rate in ml/min/1.73 m^2^ was estimated using the Haycock-Schwartz formula [24]. 

### 2.1. Testing for Microalbuminuria

Testing for microalbuminuria was then carried out on the urine specimens of all the study participants with negative proteinuria, nitrite (detectable by Combi-10), with negative microscopic findings for urinary tract infection (UTI), and results entered into the individual's study proforma. The Micral test (Micral test II) strips have a reported sensitivity and specificity of 100% and 91%, respectively [9]. Each test strip was dipped into the urine specimen for five seconds (timed with a stop watch) such that it did not touch the side of the specimen container during the process. The Micral test strip was placed across the top of the urine specimen container and result read after one minute, using the manufacturer's guidelines. The intensity of the colour produced was considered proportional to the albumin concentration in the urine. There were four different colour blocks on the Micral test strip container (corresponding to 0, 20, 50, and 100 milligram per litre i.e., mg/L) corresponding to the varying levels of albumin concentration in the urine specimen. A reading of 20 mg/L and above was considered as positive for microalbuminuria. The results were immediately entered into the individual's study proforma. 

Single sample was collected each for all the tests done during the study (early in the morning). All study participants with detected microalbuminuria, and those excluded from the study with evidence of UTI were counseled and subsequently referred with their results to the Paediatric Nephrology Clinic and Sickle Cell Clinic respectively for further evaluation, treatment, and followup. The haemoglobin genotype results of controls were communicated to them after the study.

## 3. Data Analysis 

Data was analyzed using the SPSS version 15.0. Data were analyzed quantitatively and presented in the form of frequency tables and proportions. The chi-square test was used to evaluate the significance level between proportions while the student *t*-test was used to determine the statistical significance of mean values. Further statistical analysis using bivariate correlation analysis and multivariate regression model were also carried out. A *P*-value less than 0.05 was considered as significant.

## 4. Results

Of the 200 subjects evaluated 109 (54.5%) were males while 91 (45.5%) were females. The controls comprised of 105 (52.5%) males and 95 (47.5%) females. There was no significant difference in the gender distribution of the study population (*P* = 0.688). The mean age of the subjects was 11.2 ± 3.8 years while that of the controls was 11.3 ± 3.8 years (range 4–17 years). The subjects and controls were well matched with respect to their age (*P * = 1.000). The social class of the subjects and controls ranged from class 1 to 5 as shown in Table 1. The mean body mass index (BMI) for all subjects was smaller (15.9 ± 1.9 Kg/m^2^) compared to the controls (17.8 ± 2.6 Kg/m^2^). Similarly the difference in the mean BMI between the subjects and controls was statistically significant (*P* = 0.000).

Thirty seven (18.5%) of the 200 subjects had microalbuminuria as compared to 5 (2.5%) of the controls that also had microalbuminuria (*P* = 0.001) as depicted in Table 2. There was a slight female preponderance (19.8%) compared to the males (17.4%) *P* = 0.70 as shown in Table 2.

Sixty-six (33% of the subjects were less than ten (4–9) years of age while 134 (67%) were ten years and older (10–17 years). In the age group less than 10 years, the prevalence of microalbuminuria was 9.1% as against 23.1% in those subjects who are ≥10 years of age. The mean age of the study subjects with microalbuminuria was 11.6 ± 4.2 years while those without microalbuminuria were 11.6 ± 3.7 years. Consequently, the difference in the mean age among the subjects was significant (*P* = 0.016). It was also found that among subjects aged four years, two (2) of the participants (i.e., 18.2%) had microalbuminuria in their early morning urine. 

Comparison of the various social classes (from 1 to 5) among subjects with and without microalbuminuria showed statistical significance in social classes 4 and 5 (*P * = 0.002 and 0.001, resp.) whereas the comparison of social classes 1 to 3 did not reveal any statistical significance as shown in Table 2.

Thirty-one (83.8%) of the subjects with MA had experienced previous hospitalizations following painful crises. Of the 31 subjects with MA, 4 (30.8%) have had one episode of painful crisis while 7 (63.6%) and 20 (83.3%) have had two and ≥3 episodes of painful crises, respectively, necessitating hospitalization(s). The difference was statistically significant (*P* = 0.001) as shown in Table 2. Also six subjects (3.9%) with microalbuminuria did not experience any painful crises requiring hospitalization in the preceding two years.

The mean number of blood transfusions in the subjects with microalbuminuria was 1.9 ±1.2 times as against 1.5 ± 0.9 times in subjects without MA. The difference was not statistically significant (*t* = 2.28; *P* = 0.230). The mean haemoglobin level in subjects with MA was 7.2 ± 1.1 g/dl while it was 7.8 ± 1.1 g/dl in subjects without microalbuminuria, and the difference was statistically significant (*t* = 3.49; *P * = 0.002).

There was no significant statistical relationship between the number of blood transfusions in the past, anthropometric characteristics such as weight, height, and BMI. The mean systolic blood pressure (BP) in the subjects with and without microalbuminuria was 93.8 ± 9.8 and 94.8 ± 8.6 mmHg, respectively, while the mean diastolic components were 60.3 ± 1.2 and 60.9 ± 6.2 respectively. Similarly there was no significant statistical relationship between microalbuminuria and systolic blood pressure (*t* = 0.62; *P* = 0.600) and diastolic BP (*t* = 0.53; *P* = 0.600), respectively. 

Also there was no significant difference between the mean serum creatinine of the subjects with microalbuminuria and those with negative microalbuminuria (*t* = 0.29; *P* = 0.769). Similarly, the difference in glomerular filtration rate (GFR) between subjects with and without microalbuminuria was not statistically significant (*t* = 0.96; *P * = 0.337). In all, the serum creatinine and GFR of the study subjects were within normal limits.

However, significant statistical relationship existed between microalbuminuria and age of subjects (*P * = 0.016), number of painful episodes requiring hospitalization (*P * = 0.001), and haemoglobin levels (severity of anaemia), *P* = 0.002. In the control group, BMI was the only characteristic that showed significant statistical relationship with microalbuminuria (*t* = 2.63; *P * = 0.009).

On further statistical analysis using multivariate regression model both haemoglobin levels, and number of previous hospitalizations following painful crises in the study subjects remained statistically significant. However, only haemoglobin levels among study subjects showed statistical significance with microalbuminuria using bivariate correlation analysis (see Table 3).

## 5. Discussion

The prevalence rate of microalbuminuria (MA) of 18.5% obtained in this study is comparable to the 18.4% recorded in a Jamaican study [10] but lower than the rates of 19.2%, 20.3%, and 26.5.3% reported from India [25], Benin- City, Nigeria [13] and United States of America (USA) [12], respectively. It is not very certain why the differences exist in the prevalence rates of microalbuminuria between the studies. Different *β*-s haplotypes prevalent in different races and age variation in the study subjects as well as methodology applied for screening microalbuminuria may be partly responsible. The rate of MA was found to be higher among subjects aged >10 years in the current study. Similar observation has been reported by other investigators [7, 10, 11]^.^

Quantitative assay of microalbuminuria employing double-antigen radioimmunoassay in timed urine has an edge over the conventional urinalysis dipstick on spot urine in the sensitivity and specificity in microalbuminuria determination [9]. As with this study, urinalysis dipstick (Micral test strip) was employed in the Indian [25] as well as Benin [13] studies respectively. Unlike the latter study confounding variables for proteinuria like diabetes mellitus and retroviral infection were excluded in the current study. The prevalence rate of microalbuminuria among the controls is 2.5%.

Microalbuminuria was recorded in subjects aged as low as 4 years similar to the findings by Imuetinyan and colleagues in Benin City [26] where microalbuminuria was recorded in preschool children but at variance with the USA study [12] where MA was absent in under seven subjects. Presence of microalbuminuria in younger age group in the current study may be an index of severity of sickle cell anaemia in our environment (probably due to the frequency and severity of painful crisis following exposure to recurrent infections including malaria) other than age alone. It is known that while inheritance of a single abnormal haemoglobin genes (sickle cell trait) may protect against malaria, inheritance of two abnormal haemoglobin gene does not confer any such protection and malaria is a major cause of ill health in children with sickle cell anaemia [27, 28]. There is now increasing evidence that malaria not only influences outcome but also changes the manifestation of sickle cell anaemia in Africa [28].

Though microalbuminuria occurred more is in females (19.8%) than males (17.4%), this was not significant. This finding is in consonance with the observation by Dharnidharka and colleagues [12]. It is not very certain why the higher prevalence of microalbuminuria in females. Female children in general as well as those with sickle cell anaemia have a higher frequency of urinary tract infection [12] that could result in macroalbuminuria. However in the current study urinalysis and urine microscopy were employed to exclude such subjects as in other similar studies. It could be possible that genetic influence may be partly responsible. Further studies are required to ascertain the true possibility.

The prevalence of microalbuminuria was higher in subjects of low social background. Poverty and ignorance may be possible reasons why subjects of low socioeconomic background may not have equal access (in terms of their health knowledge and ability to pay for health services) to good basic health care compared to their counterparts of higher social status resulting in higher proportion of sickle cell morbidity [29, 30] including microalbuminuria in them compared to subjects from higher socioeconomic backgrounds who are more likely to practice better healthy living. The low socioeconomic class has low health seeking behavior and some may even present late to hospitals usually after patronizing nonorthodox care centres without remarkable improvements. This increases the morbidity and mortality in them.

The current study found a significant relationship between previous history of hospitalization(s) following painful episodes as an indicator of disease severity and microalbuminuria. This is at variance with observations of other workers [7, 10, 12]. This observation suggests that vasoocclusion, glomerular hyperfiltration and even the proposed haemolysis associated vasculopathy may be responsible for the increased urinary albumin excretion rather than pathogenic mechanisms in children with sickle cell anaemia [10].

However, renal function (serum creatinine) was preserved in the subjects in spite of the detection of microalbuminuria in some cases with no correlation between MA and serum creatinine or GFR in the subjects. This has been previously reported by other workers [11, 31]. It is known that serum creatinine as a clinical marker of renal function is not a reliable indicator of early stage glomerulopathy in sickle cell disease because of increased glomerular filtration rate, lower muscle mass, and increased tubular secretion of creatinine in individuals with sickle cell disease [32]. Therefore the detection of microalbuminuria seems to be an important marker of early glomerular injury in patients with sickle cell anaemia.

Lower haemoglobin levels were significantly associated with MA in children with sickle cell anaemia in this study as have been reported by other workers [7, 33]. In contrast to this observation, Alvarez et al. [11] as well as Aoki and Saad [34] working differently did not document any association between MA and haemoglobin concentration in their series. This observation in the current study may be due to chronic sickling phenomenon in the kidneys owing to medullary hypoxicischaemia which is central to sickle cell nephropathy [1] as evidenced by presence of microalbuminuria in them. 

In conclusion, microalbuminuria occurs in a significant number of children with sickle cell anaemia. Increasing age, lower haemoglobin concentrations, and higher number of hospitalizations following painful crises were found to correlate positively with microalbuminuria. Hence, considering the significance and proven role of microalbuminuria in the detection of early renal impairment in other disease conditions like diabetes mellitus and hypertension, it is recommended that screening for MA should be incorporated into the management of sickle cell anaemia children with associated risk factors for MA. Such measure will assist in early detection and improve case management of a child with sickle cell anaemia creating an opportunity for the employment of appropriate intervention therapy.

Some limitations have been observed in the current study. Screening microalbuminuria in three serially collected urine samples over 2- to 3-month period with two or three measurements positive for microalbuminuria would have provided better results in terms of persistent microalbuminuria. This is because some patients may have sporadic microalbuminuria while others have persistent MA. Also, the cross-sectional design of the current study does not allow for the determination of the full significance of paediatric microalbuminuria in the development of kidney disease. Studies that would followup the subjects well into adulthood would help to determine the true predictive value of childhood microalbuminuria. 

Also we relied on the clinical history obtained from caregivers when such information was not available in the case record files of the subjects bearing in mind the influence of recall bias.

## Figures and Tables

**Table 1:** Demographic characteristics of subjects and controls.

Variable	Subjects *n* (%)	Controls *n* (%)	*χ* ^2^	*P* value
Age group (years)				
4–8	54 (50.0)	54 (50.0)		
9–13	82 (50.0)	82 (50.0)	0.001	1.000
14–17	64 (50.0)	64 (50.0)		
Gender				
Male	109 (54.5)	105 (52.5)	0.16	0.688
Female	91 (45.5)	95 (47.5)		
Social class				
1	18 (9.0)	25 (12.5)		
2	27 (13.5)	46 (23.0)		
3	42 (21.0)	49 (24.5)	12.296	0.015
4	95 (47.5)	68 (34.0)		
5	18 (9.0)	12 (6.0)		

**Table 2:** Age, gender, social class, and number of hospitalizations of subjects and controls with and without microalbuminuria.

Variables	All subjects [*n* = 200]				All controls [*n* = 200]			
	With MA	W/OMA	*χ* ^2^	*P*-Value	With MA	W/OMA	*χ* ^2^	*P* value
Microalbuminuria	37 (18.5%)	163 (81.5%)			5 (2.5%)	195 (97.5%)		
Age (in years)								
4–8	6 (11.1)	48 (88.9)	22–68	0.001	0 (0.0)	54 (100.0)	3.74	0.154
9–13	7 (8.5)	75 (91.5)			2 (2.4)	80 (97.6)		
14–17	24 (37.5)	40 (62.5)			3 (4.7)	61 (95.3)		
Gender								
Male	19 (17.4)	90 (82.6)	0.182	0.70	2 (1.9)	103 (98.1)	0.01	0.96
Female	18 (19.4)	73 (80.6)			3 (3.2)	92 (96.8)		
Social class								
1	5 (27.8)	13 (72.2)	1.113	0.288	1 (4.0)	24 (96.0)	0.26	0.607
2	6 (22.2)	21 (77.8)	0.29	0.592	0 (0.0)	46 (100.0)	0.49	0.266
3	8 (19.0)	34 (39.0)	0.01	0.918	2 (4.1)	47 (95.9)	0.08	0.772
4	9 (9.5)	86 (90.5)	9.78	0.002	1 (1.5)	67 (98.5)	0.04	0.848
5	9 (50.0)	9 (50.0)	13.02	0.001	1 (8.3)	11 (91.7)	0.09	0.762
Number of Hospitalizations								
(in the preceding 24 months)								
None	6 (3.9)	146 (96.1)	88.915	0.001				
Once	4 (30.8)	9 (69.2)						
Twice	7 (63.6)	4 (36.4)						
≥3	20 (83.3)	4 (16.7)						

Key—With MA: with microalbuminuria; W/O MA: without microalbuminuria.

**Table 3:** Bivariate correlation analysis of the dependent variables of microalbuminuria among subjects.

Dependent variables	Correlation coefficient (*r*)	Significance
Age (years)	−0.046	0.516
Gender	0.013	0.856
Social class	0.077	0.270
Previous hospitalization	−0.080	0.263
Systolic blood pressure	0.045	0.533
Diastolic blood pressure	0.037	0.607
Hemoglobin level	0.221	0.002
Serum creatinine	0.020	0.780
Glomerular filtration rate	−0.068	0.340
